# Menstrual management in transgender and gender diverse individuals: psychiatric and psychosocial considerations

**DOI:** 10.3389/fpsyt.2024.1422333

**Published:** 2024-10-29

**Authors:** Arslaan Arshed, Sharon Madanes, Stephanie Pottinger, Marra G. Ackerman, Allison B. Deutch

**Affiliations:** Department of Psychiatry, New York University, New York, NY, United States

**Keywords:** menstruation, transgender, transmen, mood disorder, gender diverse, menstruator, gender dysphoria

## Abstract

Transgender and gender-diverse (TGD) menstruators are individuals assigned female at birth (AFAB)*, who retain the capacity to menstruate and have a gender identity that differs from their natal sex. Reports indicate up to 1.6 million individuals in the US identify as TGD. Until recently, the mainstream menstrual discourse has failed to capture the experience of transmenstruators. However, a better understanding of the menstrual experiences of TGD-AFAB will allow for more individualized patient-centered care. In this review, we provide the relevant data necessary to inform the psychiatric management of menstruation in TGD-AFAB individuals, including experiences of menstruation, preferences for menstrual management, and the impact on mental health. Our review indicates that menstrual care in TGD patients must be tailored to the individual; clinicians should remain open-minded to the unique experience of transmenstruators; gender-affirming menstrual care is necessary to reduce psychological burden. It should not be assumed that TGD-AFAB menstruators are utilizing appropriate contraceptive methods and should receive contraceptive and fertility preservation counseling. We highlight the importance of having these conversations early in the reproductive arch, even before puberty onset. Keeping in mind the gender minority stress model, in the upcoming sections, we discuss the limited body of literature on mood disorders in TGD-AFAB individuals who menstruate, undergo menstrual suppression, or continue to ovulate. The psychological impact of hormonal therapies is also reviewed.

## 1 Introduction

Earlier estimates based on population-based surveys approximated that 0.3-0.6% of the United States population, or 1 million people, identify as transgender; however recent reports indicate that figure may be closer to 1.6 million (1, 2). Similarly, the DSM-V documents the prevalence of gender dysphoria as 0.005-0.014% for those assigned male at birth, and 0.002-0.003% for adults assigned female at birth, with recent reports indicating increasing numbers (3). Still, there is a lack of familiarity and competency around the medical and mental health needs of TGD individuals. According to the 2022 US Transgender Survey (USTS), amongst 92,329 respondents, 38% identified as nonbinary, 35% as transgender women (transwomen), and 25% as transgender men (transmen) (4). Of those respondents, 24% did not seek medical care, and 48% reported at least one negative experience due to their gender identity, including being misgendered; being refused care; being addressed with harsh or abusive language; or being subjected to physically rough treatment (4). Seelman et al., 2020 found that in the 2015 USTS, 54% of transmen found their routine healthcare providers knew very little (“nothing” or “some things”) about trans-related care (5).

Although some TGD individuals undergo medical transition, many do not (6). A social transition is when an individual changes their appearance and pronouns. A legal transition involves legally changing one’s name and documents to reflect this and their identified gender. Medical transition may involve gender-affirming hormone therapy (GAHT) and/or gender-affirming surgery. In TGD-AFAB individuals, common gender-affirming surgeries include masculinizing chest surgery (i.e., mastectomy), body contouring, and metoidioplasty, as well as non-surgical chest binding. Masculinizing GAHT in TGD-AFAB individuals is usually continued lifelong to maintain virilization independent of genital surgery. Testosterone is the mainstay of masculinization, which aims to induce secondary sex characteristics and suppress feminine physical features, and in most cases causes amenorrhea. At least 80% of TGD individuals have either taken, tried, or wanted to take GAHT at some point (7). Gender nonbinary or gender non-conforming individuals who do not identify as transgender may take hormones to develop more masculine sexual secondary characteristics. Although some gender-affirming treatments include a hysterectomy or oophorectomy, many TGD-AFAB menstruators wish to maintain their reproductive organs and childbearing potential (8–11).

Compared to other medical specialties, psychiatry has had a more fraught relationship with the TGD community. The Diagnostic and Statistical Manual of Mental Disorders (DSM) 3rd and 4th editions, for example, conceptualized gender identity disorder (GID) as pathologic. Although GID was subsequently reclassified as gender dysphoria (**Supplementary Table 2**) in DSM-V (3), literature continues to indicate a higher prevalence of mental health disorders among transgender individuals compared to the general population or cisgender individuals. TGD people experience a two-fold higher risk of receiving a psychiatric diagnosis compared to cisgender people and are diagnosed with a mood, anxiety, or psychotic disorder, with odd ratios of 1.5:1, 3.9:1, and 3.8:1 respectively (12). These disparities can be understood through the minority-stress framework, which describes the complex interaction between majority (cis-gender) and minoritized groups (transgender and nonbinary) communities.

Building on this, we aim to provide a comprehensive review of the experiential and clinical data necessary to inform the care of TGD-AFAB individuals. We review the gendered history of menstruation; the value of gender-inclusive menstrual dialogue; guidelines for transgender health around menstrual suppression, fertility preservation, and contraceptive counseling; and elucidate the interplay between gender dysphoria, menstruation, and hormone-related mood disorders in TGD-AFAB individuals.

## 2 Methods

We searched PubMed from March to July 2024 without date limitations for studies of TGD menstruators. We did not utilize any language restrictions and included historical and contemporary terms related to gender identity such as “transgender”, “transgender diverse”, “transmen”, “menstruators”, “assigned female at birth”, “nonbinary”, and “gender diverse”. We utilized similar terms through Google to locate magazine articles, books, government announcements, and websites.

## 3 Historical and social considerations

Menstruation is a biological process that is steeped in historical significance. In Greek mythology, menstrual blood is referred to as “the supernatural red wine,” conferring “miraculous power” to the gods (13). In Ancient Rome, menstruators were also thought to have “great powers,” and menstrual blood was recommended as a treatment for illnesses of all kinds, including rabies, malaria, and epilepsy (13). Many indigenous tribes emphasize the strength of menstruators and menstruation itself. The Cherokee Nation of Oklahoma believes that menstruation is powerful, a source of feminine strength, and a destructive force against evil and enemies (14). In Cherokee mythology, *Stoneclad*, a cannibalistic monster was undefeated by warriors until he was in the presence of seven menstruating virgins who took his strength away (13). To channel the power of menstruators, Cherokee men would also practice bloodletting before games and battles (14).

Menstruation has also been invoked as evidence of women’s inferiority, a notion that dates as far back as Aristotle (15). In biblical Jewish culture, menstruating women were required to remain separate from men for the duration of their menses. Orthodox Jewish women continue to use the ritual bath (mikvah) following menstruation and before engaging in intercourse after menses. The Roman Catholic Church of Thirteenth Century Europe perpetuated the “stigma of menstrual uncleanliness … [and] perceptions of menstrual blood as vile and polluting,” encouraging women to refrain from taking Holy Communion while menstruating (16). In Islamic culture, menstruating women are unable to pray, enter mosques, or touch the Quran; they are required to clean themselves through a spiritual bath (ghusl) each time menses concludes (17). Additionally, if menstruating, they are barred from completing the final ritual of Hajj, Tawaf-ul-Ziyarah, which involves circumnavigating the Kaaba to commemorate the end of the annual pilgrimage to Mecca (17).

By suggesting that menstruation was not a signifier of shame or weakness, but rather something that could be “managed,” the feminist movement of the 20^th^ challenged the historical menstrual and reproductive rhetoric and through the lens of women’s empowerment, moved conversations around menstruation into the mainstream (18). More recently, conversations around menstruation have come to encompass an even wider range of perspectives, including those of transgender and non-binary menstruators. Kosher et al. (2023) refer to this as “the plurality of menstrual experiences,” encapsulating the increasingly common viewpoint that “not all women menstruate, and not all menstruators are women’’ (19, 20). Said differently, menstruation is a deeply nuanced and subjective experience for TGD-AFAB individuals (21, 22).

While the experience of menstruation among TGD individuals is conventionally understood as “the individual who was AFAB; identifies with masculinity; experiences intense dysphoria around menstruation because it is gendered feminine; and is actively undergoing intervention to suppress or eliminate menstruation,” the reality is much more complex (19). Fortunately, data examining TGD-AFAB individuals’ attitudes toward menstruation, considerations around menstrual management, and decisions about menstrual suppression are emerging. Chrisler et al. (2016) surveyed 150 TGD-AFAB individuals to examine attitudes toward menstruation and menstrual suppression (21). While some respondents expressed a positive view of menstruation, the majority reported “mixed or ambivalent attitudes.” Half of the participants reported that they would elect to suppress menstruation if suppression were possible without the use of testosterone. Fifty percent also agreed that “not menstruating would enhance their sense of their masculine identity” (21). More recently, Kosher et al. (2023) identified three major themes pertaining to menstruation in the TGD population: 1) dysphoria; 2) tensions between femininity and masculinity; and 3) transnormative pressures (19). A 2023 study by Schwartz et al. examined TGD adolescents’ (N=129) experiences of menstruation and found that a majority (93%) experienced menstrual-related dysphoria (23). A substantial number (88%) were interested in menstrual suppression, with the primary reason being a desire for a complete cessation of menses (97%) and the second to improve menstrual-related dysphoria (63%) (23).

Beyond menstrual bleeding itself, the *management* of menstruation can be a source of distress for TGD menstruators (24). Although femininity is neither a predicate for menstruation nor an outcome of menstruation, terms like “feminine hygiene product” or “feminine care” abound in the marketing and sale of menstrual products. Furthermore, TDG-AFAB individuals may experience aversion to vaginal penetration (21), and many menstrual products are designed for insertion into the vaginal opening. In 2016, the Thinx period product brand released its Boyshort style underwear with a series of video and print advertisements featuring trans male model, Sawyer DeVuyst. According to Miki Agrawal, Thinx’s Chief Executive Officer at the time, the Boyshort was “specifically designed with the trans male menstruating community in mind” and a deliberate step by Thinx to be part of “shifting [public] perspective” on the needs of the trans community (25). The introduction of the Boyshort to the period product market also came on the heels of a major shift in Thinx’s marketing strategy. Moving away from its original *For Women with Periods* advertising campaign, Thinx opted for a revised *For People with Periods* tagline. The goal, Thinx says, was an acknowledgment that menstruation “is not a trait of, nor a defining factor of, a specific gender. It is something that can occur amongst all people’’ (26).

However, the lack of inclusive period products is just one way in which TGD-AFAB individuals have been impacted by a menstrual landscape designed almost exclusively for cis-gender women. The issue of period poverty for menstruators is an underrecognized public health issue. Broadly, period poverty refers to a lack of access to menstrual education, menstrual products, and facilities that allow for proper menstrual hygiene and disposal of menstrual products (27). It also refers to “the increased economic vulnerability menstruating people face due to the financial burden posed by menstrual supplies….[and] costs accrued from pain medication and underwear used during the menstrual cycle (28). “ Period poverty also contributes to the cycle of generational poverty by adding to the marginalization of menstruators in the form of missed days of work, missed days of school, and diversion of monthly income to meet menstrual needs. TGD-AFAB communities are particularly vulnerable to the impact of period poverty. According to the University of Wisconsin-Madison Institute for Research on Poverty, nearly 34% of transmen and 24% of gender nonbinary individuals live in poverty, compared to 16% of cis-gendered individuals (29). In the 2022 USTS, TGD individuals reported facing substantially higher rates of housing instability compared to the general population (4).

To address issues of period poverty and menstrual inequity, at least 30 states and Washington D.C. have passed a combined total of 60 bills designed to improve access to menstrual health resources. While laws differ by state, they are generally aimed at improving access and affordability by eliminating sales tax for menstrual products and ensuring free menstrual products are available to individuals in public schools, correctional facilities, and homeless shelters (30). However, menstrual product availability in male-designated restrooms has become a topic of controversy in political and public discourse. Events that took place in a Connecticut (CT) High School made national headlines in 2024 after a tampon dispenser, affixed to the wall of a boys’ restroom, was torn down. The tampon dispenser was placed in the boys’ restroom in response to a relatively new CT state law that mandates public schools to provide free access to menstrual products for students. According to The Connecticut Department of Health “[t]he law was designed to affirm all genders in making empowered decisions about their health, including menstrual health. As a result, the law requires products to be provided in all-gender bathrooms, one men’s restroom per school, and all women’s restrooms in the building” (27).

However, such policies have not been applied uniformly across the United States. Nearly 47% of respondents in the 2022 USTS had thought of “moving to another state because their state government considered or passed laws that target transgender people for unequal treatment (such as banning access to bathrooms, health care, or sports), and 5% of respondents had moved out of state because of such state action” (4). In 2023, Florida signed House Bill 1521, barring TGD individuals from using locker rooms or public bathrooms consistent with their gender, highlighting continued barriers to menstrual management (31).

Unsurprisingly, research suggests that a majority of TGD-AFAB individuals feel “unsafe” or “very unsafe” managing menstruation in men’s restrooms (21). The risk of being ‘outed’ by the use of individually wrapped menstrual products (tampons, pads) appears to contribute, in part, to the preferential use of extended-use period products like menstrual cups, adult diapers, or period underwear (21). A study by Lane et al. (2022) also identified public restroom design as a “significant barrier to menstrual management” (32). Participants described large gaps between stalls, the “reflective gaze” of the mirrored sinks, high urinal-to-stall ratios, and lack of disposal sites for menstrual products as being some of the most fraught aspects of queer menstruation.

## 4 Applying consensus guidelines in gender-affirming medical care

A professional body spanning disciplines and geographic regions, the World Professional Association for Transgender Health (WPATH) tasks itself with promoting evidence-based care, education, research, public policy, and respect for transgender health. WPATH is associated with 7 regional organizations: United States, European, Canadian, Asia, Australia, Aotearoa (New Zealand, and Southern Africa. Since the organization’s formal inception in 1979, it has iteratively published standards of care (SOC). These documents have served as consensus guidelines and best practice recommendations for professionals caring for transgender and gender-diverse populations throughout the world (33). Within the last few years, there has been a shift in European countries with guideline implementation. The National Health Services England released the CASS Report in 2024, closed its Gender Identity Development Service, and recently banned puberty blockers for adolescents (34). Sweden, Finland, and Denmark continue to follow similar patterns.

While experiences of and feelings about menstruation among TGD-AFAB individuals are diverse, some may experience an exacerbation of incongruence between gender identity and perception of their bodies during menses. To address this, the most recent WPATH SOC-8 contains recommendations regarding the suppression of menstruation in adolescent and adult TGD-AFAB individuals (33). In most, GAHT with exogenous testosterone at adult physiologic doses will precipitate amenorrhea due to the sex steroid’s effect on the endometrium, and resultant endometrial atrophy (35). However, menstrual suppression with GnRH agonists, combined estrogen-progestin, or progestin-only methods can be indicated, especially in adolescent populations.

WPATH recommends considering menstrual suppression agents in adolescents only once they have reached the early stages of puberty. The Endocrine Society Guidelines from 2017 also recommend against puberty-blocking and GAHT in pre-pubertal children (36). Adolescents may wish to halt puberty to further explore gender expression and identity, take time to consider GAHT initiation with testosterone, or use adjunctive agents for breakthrough bleeding during the early stages of initiation of GAHT. Furthermore, if there is a delay in consensus about GAHT among the individual, provider, and/or guardian, menstrual suppression should be considered as an initial step (33). Engaging TGD-AFB individuals in conversations about menstrual suppression can also provide a forum to explore readiness for gender-affirming treatment and any associated mental health needs (37).

The choice of menstrual suppression agent (between GnRH agonist and progestin-only or combined estrogen-progestin methods more commonly used as contraceptive agents) is dictated largely by the developmental status of the individual, consideration of individual medical risk factors (specifically venous thromboembolism in estrogen-containing methods), potential dysphoria caused by estrogen-containing agents, cost, and availability. While GnRH agonists can delay further progression of puberty and cause the regression of some early secondary sex characteristics, these agents are costly, and not always covered by insurance (33). In cases where GnRH agonists are not reasonably available, progestin agents (available as depot injection, oral pill, intrauterine device, subdermal implant) or combined estrogen-progestin agents (available as oral pill, transdermal patch, vaginal ring) can be used. Between 55-97% of transmasculine adults achieve amenorrhea within the first 6 months of testosterone therapy (38, 39). Lower rates of amenorrhea have been described in TGD-AFAB adolescents using testosterone (59% at 6 months) and higher success rates with NETA or DMPA monotherapy (40). Breakthrough bleeding requires clinical investigation to determine etiology and most commonly requires adjustment of testosterone dosage or additional hormonal intervention. Compared to individuals who initiated treatment with GnRHa at a later age, those who initiated at an earlier age (average age 11) had lower rates of depression, anxiety, suicidal ideation, and suicide attempts (41). 

Ultimately, a third of TGD-AFAB individuals surveyed desired a hysterectomy, which would obviate the need for pelvic examinations and stop menses (5). TGD-AFAB individuals may seek a hysterectomy in response to pelvic pain (42). While pelvic pain has a prevalence of 25% among cis-gender women, higher rates have been reported in adult trans cohorts (43). In a 2020 cross-sectional survey of 486 AFAB trans participants, 72.2% reported pelvic pain after initiation of GAHT with testosterone, with most pain localizing to the suprapubic region and being characterized as “crampy” in nature (44). Additionally, this study found an association of pelvic pain after initiation of testosterone therapy with a personal history of PTSD, continued menstruation, and experiences of pain with orgasm (44). In another survey conducted between 2015-2017, 69.4% of respondents reported abdominopelvic pain specifically onset since initiation of GAHT (45). All respondents who reported curative treatment of this pelvic pain identified hysterectomy as the treatment method (43, 45).

## 5 Contraception and fertility preservation

WPATH and the Ethics Committee of the American Society for Reproductive Medicine (ASRM) recommend discussing fertility preservation and family planning before initiation of GAHT (46). The impact of testosterone on fertility depends on duration and timing of use; if started before puberty and concurrently with puberty blockers, the individual may never develop reproductive function. If GAHT is initiated after puberty, reproductive function can be restored. The use of exogenous testosterone can have lasting effects on fertility (43). While testosterone does not affect ovarian reserve, testosterone can induce gonadal suppression through its action on the hypothalamic-pituitary axis, preventing ovulation and follicle development (44). Ovarian exposure to exogenous testosterone can cause ovarian capsule thickening, which can also impair fertility (45). While most TGD-AFAB individuals on testosterone will achieve amenorrhea and ovarian suppression, it is still possible to ovulate and become pregnant while amenorrheic (47). A study of TGD-AFAB individuals on testosterone found evidence of breakthrough ovulatory events by monitoring hormonal levels.

Studies have found that a majority of TGD-AFAB individuals express parental desire and that a quarter fear infertility (8, 10, 11). Fertility preservation options include embryo, oocyte cryopreservation, and experimental ovarian tissue banking, which can be used before gender-affirming surgery or the initiation of GAHT regardless of puberty status (43). Many TGD-AFAB individuals choose to forgo fertility preservation to avoid delaying GAHT. A majority also report fears that hormone injections and the pelvic procedures required for egg retrievals will worsen gender dysphoria (9, 23, 48). Overall, utilization of fertility preservation remains low in TGD communities. In a survey of TGD menstruators referred to a community health clinic in Tel Aviv, Alpern et al. found that while 61.9% wanted a child, only 5.8% accessed fertility preservation (9). There are many barriers to access to fertility preservation, including the high out-of-pocket expense of cryopreservation and assisted reproductive technologies and lack of adequate insurance coverage; provider discomfort in discussing fertility preservation with TGD-AFAB patients; fears of discrimination for the transgender parent as well as the child; and legislation barring gender-affirming care (9, 11, 35, 43).

Among TGD-AFAB individuals, 30% of pregnancies are unintended (47). Therefore, discussing contraception options is imperative, especially given the potential teratogenicity of testosterone. The teratogenic risks of testosterone in animal studies are well documented; however, the literature on human outcomes is sparse. *In utero* exposure to testosterone can result in abnormal development of genitalia including labial fusion and clitoromegaly, and several hypotheses that these exposures contribute to anorexia nervosa and autism in offspring (49). However, a longitudinal pregnancy cohort study failed to confirm this association (50). In a sample of 25 TGD-AFAB individuals who were on testosterone prior to pregnancy, 20% were on testosterone for at least some portion of their pregnancies. Among this sample size, rates of fetal anomalies, developmental disorders, or disorders of sexual development were equivalent to rates in TGD-AFAB individuals not on testosterone (47). More research is needed to identify long-term neurodevelopmental impacts of perinatal testosterone exposure.

Contraception in this specific population is inconsistently reported; however, estimates are between 20-60% (51). Contraceptive methods may be underutilized due to several factors, including the lack of education provided by medical providers, lack of access, as well as the frequent misconception by both transmen and providers that testosterone provides reliable contraception (51, 52). Furthermore, the irregular nature of ovulation while on GAHT may make pregnancy harder to anticipate; this places TGD-AFAB menstruators at high risk for unplanned pregnancy, especially coupled with limited access and inconsistent contraceptive use. And just as menstruation can worsen gender dysphoria by the repetitive reminder of natal sex, contraceptive methods, and fertility preservation may too (48). Oral Contraceptive Pills (OCPs) are marketed to women and can serve as daily reminders of anatomy; longer-term options, such as IUDs are invasive, painful, and require a pelvic exam and manipulation, exposing transmen to dysphoria-based discomfort. Other barriers to contraceptive use include concerns that hormones from OCPs can counteract the effects of testosterone by increasing sex hormone-binding globulin which binds to testosterone (51).

## 6 Psychiatric and psychosocial considerations

In understanding mood disorders among TGD-AFB individuals, we consider biological and psychosocial contributors. Biologically, GAHT with exogenous testosterone in TGD-AFAB individuals is known to increase total brain volume, total gray matter volume, thalamus volume, and cortical thickness, specifically in frontal and parietal brain areas (46, 47, 52). Kiyar et al. (2022) found that the neural profile of TGD individuals assigned male at birth (AMAB) shifted from natal sex to their gender identity after 6-10 months of GAHT on fMRI. This confirmed that total testosterone is involved with the neural processing of emotions (51). However, it remains unclear how testosterone-driven neural profiles change with menstrual-related mood disorders.

The gender minority stress model outlines psychological processes that ultimately lead to mental health conditions and mood disorders (49). Chronic exposure to gender-based rejection; gender victimization; lack of gender affirmation; violence targeting TGD individuals; and legislation barring gender expression leads to the internalization of negative self-concept, fosters negative anticipation of upcoming experiences, and delays engagement in gender-affirming care. Internalization of negative self-concept may also contribute to gender dysphoria and a variety of mental health conditions (49).

### 6.1 Premenstrual disorder and premenstrual dysphoric disorder

There is limited data on premenstrual disorders amongst TGD-AFAB individuals as this population is often excluded from studies about menstruation (50). PMD symptoms are categorized as predominantly physical, emotional, or mixed (48). The prevalence rate of PMD among cisgender women is estimated at around 20-40 %, with around 5% of cisgender women experiencing PMDD, a severe form of PMD that was added to the DSM-V in 2013 (48, 53). An exclusion criterion of PMDD is an exacerbation of an underlying psychiatric disorder, which can include gender dysphoria. Undoubtedly, comorbid gender dysphoria and the inherent worsening of dysphoria for many TGD-AFAB individuals with the onset of menses complicates the diagnosis of underlying PMDD (50). Despite diagnostic challenges, anyone with a menstrual cycle, including a TGD-AFAB person, is susceptible to PMD and PMDD.

Treatment for PMDD, which often includes cycle suppression, can dovetail with gender-affirming care. Low-dose selective serotonin reuptake inhibitors (SSRIs), with either luteal or continuous dosing, remain a first-line treatment option (48, 54). Other treatments include herbal supplements, such as chasteberry which is supported by data from a systematic review, and vitamins, including Vitamin B6, Calcium, and Magnesium (48, 55).

People with PMDD are also at high risk for suicidality; a recent systematic literature review found that 7-16% of people with PMDD have attempted suicide (56) compared to 0.7% of the general population in the most recent CDC data. Given the high baseline rate of suicide attempts (between 32-50%) in transgender people (57), screening for PMDD and suicidality in TGD-AFAB menstruators merits increased clinical attention (58, 59). At the time of this writing, we could not find data on the prevalence rates of PMDD among TGD-AFAB menstruators.

### 6.2 Depression and anxiety

There is limited literature on depression and anxiety among transmen, as most studies combine transgender identities. In a review of the literature published between 2013-2018, Nguyen et al. (2018) found lower depressive symptoms in TGD-AFAB individuals receiving GAHT across all studies (60). They also found that transgender men and women over the age of 50 receiving GAHT experienced a reduction of anxiety symptoms, as well as decreased cortisol awakening response and perceived stress (60). Although they found ongoing self-reports of high stress, 70 TGD individuals had a decrease in cortisol awakening response, which has been linked to decreased anticipation of stress (61, 62).

### 6.3 Post-traumatic stress disorder, post-traumatic stress symptoms and discrimination

TGD individuals report higher rates of exposure to traumatic events, including abuse in childhood, physical assault, intimate partner violence, and sexual assault (63–65). They also experience pervasive discrimination, discrete traumatic events linked to discrimination, and anti-TGD bias (66). In a national sample, up to 63% of TGD individuals reported discriminatory events leading to eviction, job loss, losing stable housing, denial of medical care, or losing an intimate relationship (67). Despite this, the wide range of PTSD prevalence within the TGD community suggests that there may either be varying detection strategies for PTSD or an over-pathologizing of responses to trauma and discrimination. Studies linking symptoms to specific traumatic events found lower rates of PTSD diagnosis when compared to studies that assessed PTSD symptoms generally (64, 65, 68, 69). Given this, it is important to delineate trauma from discrimination in TGD individuals, as conflation of the two may result in over-pathologizing responses from discrimination-based psychological distress (66).

### 6.4 Bipolar disorder, mania

There is limited to no data to guide the management of bipolar affective disorder in patients undergoing GAHT. Studies that associate manic symptoms with exogenous testosterone are often reported in cisgender men with supraphysiologic levels of anabolic androgenic steroids (70, 71). This contrasts with GAHT in TGD-AFAB individuals, where the dosing of testosterone targets physiologic levels. There is limited data on the association of mania in transmen with exogenous testosterone administration (36). Thus far, there is no data to indicate that appropriate GAHT dosing of testosterone would lead to an induction of mania, but close monitoring during testosterone initiation in at-risk individuals is warranted.

### 6.5 Psychosis

Current literature indicates an increased prevalence of schizophrenia spectrum disorders and psychosis amongst TGD individuals (72). In a review of 254 million discharges in the United States National Inpatient Sample database in 2019, Hanna et al. (2019) found that 14.5% of TGD patients were diagnosed with psychosis, compared to 4.3% of cis-gendered patients, and 14.2% of TGD patients were diagnosed with schizophrenia, compared to 2.8% of cis-gendered patients (57). In their literature review, Barr et al. (2021) found variability in the reported rates of psychotic illness among TGD individuals, which they attributed to varying recruitment strategies, racial bias in diagnosis, and demographic differences, as TGD study participants were on average 17 years or younger (72). Through a cohort study examining birth certificates and national insurance data in the Netherlands from 2006-2020, Termorshuizen et al. (2023) found that of 5,564 TGD individuals, the TGD-AFAB cohort was substantially more likely to be diagnosed with a non-affective psychotic disorder when compared to TGD-AMAB (73). They also found that up to 50% of TGD individuals were diagnosed with psychosis after the first registration of data indicative of TGD status, and up to 83.7% were documented at the same time (73). These results, coupled with the historical classification of gender dysphoria as symptomatology within the realm of psychotic spectrum illness, suggest a continued risk of diagnostic bias of psychosis amongst TGD individuals.

### 6.6 Aggression

Although WPATH SOC Version 7 listed destabilization of psychiatric disorders as a potential side effect of testosterone, this was removed in WPATH SOC Version 8 (33). There is no documented consistent association or correlation between anger and serum testosterone levels; however, if irritability, mood changes, or anger do occur, these symptoms are more likely during the first year of initiation of GAHT (74, 75). Of the literature linking anger expression with testosterone in TGD-AFAB individuals, there was no increased risk for psychiatric hospitalization or self-harm (76, 77). Self-report measures of irritability and aggression decreased after gender-affirming surgical intervention, perhaps reflecting a decrease in depressive symptom burden (78). If mood changes occur with testosterone treatment, then it may be helpful to consider dose adjustments; changing from short-acting to long-acting intramuscular injections, or changing to gel formulations (79).

### 6.7 Quality of life, body satisfaction, and self-esteem

Several studies indicate a statistically significant improvement in self-reported quality of life and self-esteem in TGD-AMAB and TGD-AFAB individuals receiving GAHT (80–82). Silve et al. (2021) interviewed 113 people (60 TGD-AMAB and 53 TGD-AFAB) using quality of life questionnaires and found that in transmen, improved quality of life was associated with employment, being in a stable relationship, physical activity, and increased body hair (83). However, in a systematic review of 32 articles looking at TGD individuals on GAHT, not all studies reported a consistent improvement in body satisfaction when cross-sectional data was analyzed. They also noted that the literature on quality of life was inconsistent, ranging from improved, neutral, and worsened quality of life (82).

### 6.8 Sexual desire

One year of GAHT treatment in TGD-AFAB individuals is associated with an improvement in sexual function, specifically masturbation frequency, arousal, sexual fantasies, and desire (78). Additionally, increased libido is also linked to the earlier treatment with GAHT (84). In a cross-sectional analysis of data from the PRIDE study in 2021-2023, TGD-AFAB individuals on GAHT reported increased sexual desire (85). They theorized that GAHT increases the quality of life and self-image, which in turn positively impacts other areas of their life, including sexual desire (85).

### 6.9 Gender dysphoria

Although the literature indicates a decrease in gender dysphoria with GAHT, long-term affirmation is also impacted by social gender recognition, discrimination, and other variables. In a cross-sectional survey of 359 TGD individuals (219 TGD-AMAB and 140 TGD-AFAB), higher levels of dysphoria were related to sociological indicators of identity (ex: legally changing sex on identification) in those utilizing masculinizing GAHT compared to those who were not, suggesting that gender dysphoria may be related to sociological indicators of identity rather than just the hormones themselves (86). In that same study, the AFAB individuals who ultimately initiated GAHT noted global gender dysphoria decreased at 3 months and subsequently increased at 6, 12, and 24 months (86). When delineated further, it seems that subjective dysphoria was decreased, but dysphoria impacted by social and sociolegal factors had increased (86). In another study from Australia, TGD-AFAB individuals undergoing GAHT were found to have a significant reduction in gender dysphoria within the first few months of treatment, however, over 2 years had an increase in dysphoria related to social and sociolegal factors (87).

## 7 Age-related considerations

Despite the expectation that the number of Americans aged 65 and older will surpass children in 2023 and an estimation of at least 700,000 senior TGD individuals, there is limited literature on older TGD individuals in the United States (88). Most data and knowledge about TGD individuals come from European cohorts, with a larger proportion documented as transgender women. Under-representation of TGD-AFAB seniors in the literature makes it difficult to determine the long-term impact of hormonal and GAHT interventions on TGD-AFAB menstruators (89, 90).

Studies on cis-gendered individuals with a prostate, and post-menopausal women on hormone replacement therapy (HRT) suggest that sex hormones can be neuroprotective and improve cognition (91, 92). Cognitive changes in menopause are believed to be related to decreased estrogen availability, and androgen receptors in the hippocampus and prefrontal cortex (93). However, it remains unclear if these studies can be extrapolated to TGD-AFAB menstruators.

Data drawn from health insurance companies and ICD codes indicates TGD individuals had a statistically significant elevated probability of being diagnosed with dementia when compared to cis-gendered individuals (94). Furthermore, TGD-AFAB individuals aged 45+ who identified as ethnically minoritized reported more memory loss and confusion in the past year when compared to their cis-gender counterparts (95). It is important to note that these studies combine transgender men and women, and often do not ask further questions about non-binary and fluid identities.

When gender identity is parsed out, the findings change. Heesewijk et al. (2023) found that transgender men had an increased risk of dementia compared only to cis-gender women; later research in 2021 found that transgender men had a similar cognitive functioning profile when compared to cisgender men and cisgender women (96). It has been theorized that the reported increased rate of dementia may be associated with delayed gender affirmation, repeated dysphoric episodes when menstruating, as well as limited social support (97).

Currently, a link between GAHT and impaired cognition has not been consistently described in the literature (97). Although there is little to no published literature on lifestyle factors impacting cognitive decline in TGD-AFAB individuals, when compared to cis-gender people, there is an overall increased prevalence of known risk factors, including higher rates of cigarette and alcohol consumption and lower physical activity (97). A higher burden of depressive symptoms may also impact cognition; rates of clinically significant depressive symptoms in older transgender individuals have been reported as high as 48%, which is pointedly higher than the general population of 6-8% (98, 99).

## 8 Conclusions

The language of TGD healthcare continues to evolve towards improving inclusivity. Medical providers should engage patients in discussions about their gender identity, how they relate to it, and what it means to them, as it can vary greatly. An example is the variability of preferred pronouns- she/her/he/him/they/them. WPATH guidelines recommend providers use active listening to encourage exploration in those uncertain about their gender identity. Rather than impose their narratives or preconceptions, providers should assist people in determining their paths. These conversations should also include the changing legislation for TGD-affirming care. **Supplementary Table 4** provides examples of how to address topics discussed in this review.

Limitations to our review include an emphasis on North American data and Euro-centric experiences. To extrapolate this to other cultures and ethnicities requires sensitivity to their specific identities and contexts. There are also significant limitations in the current global literature on TGD-AFAB individuals. Survey language often omits TGD-AFAB as a gender category or groups all TGD individuals into one category, which excludes the diversity and spectrum range of gender. This has led to limited literature on the impact of menstruation, menstrual-related disorders, pregnancies, fertility, and exogenous testosterone exposure among TGD-AFAB individuals.

Our review indicates that the WPATH SOC-8 has moved beyond a focus on surgery and hormones, now including sexual, reproductive, mental, and primary health care services. There remains a need for additional research on the reduction of barriers to OBGYN care, contraception, and fertility preservation in TGD-AFAB individuals. These barriers can be addressed through their inclusion in medical, psychiatric, and menstruation-related research, as well as in medical, graduate, and residency-level curricula. Doing so may allow more comfort in treating this increasingly prevalent patient population.
