# Supplementary material for: Menstrual management in transgender and gender diverse individuals: psychiatric and psychosocial considerations
Source: Front Psychiatry. 2024 Oct 29;15:1422333. doi: 10.3389/fpsyt.2024.1422333 (PMC11554503; doi:10.3389/fpsyt.2024.1422333)
Supplement: Supplementary file 1 [file DataSheet1.docx]

# Supplementary Material (Tables)

| **Gender inclusive language** | **Definition** |
| --- | --- |
| Sex | A category of sex organs (male, female, intersex) which are differentiated by reproductive function |
| Gender Identity | Culturally dependent social norms and roles that are associated with an individual’s natal sex |
| Menstruator | An individual who menstruates from their vagina or vaginal opening |
| Transgender | Gender identity that socially *differs* from birth sex |
| Cisgender | Gender identity that socially *aligns* with birth sex |
| Masculine Of Center (MoC) | A broader gender expression term used to describe a person who identifies or presents as more masculine than feminine |
| Nonbinary | A gender identity that does not fall within the male vs female gender paradigm, with representations that may vary across cultures |
| Two-spirit | A third gender in many indigenous communities, often called “suprabinary,” are believed to hold mystical connections |

*Table 1. Glossary of gender-inclusive languag*e and definitions (2, 5, 7, 36)

| **DSM-IV: Gender Identity Disorder (1994)** | **DSM-V: Gender Dysphoria (2013)** |
| --- | --- |
| A. A strong and persistent cross-gender identification (not merely a desire for any perceived cultural advantages of being the other sex). In children, the disturbance is manifested by four (or more) of the following:   1. Repeatedly stated desire to be, or insistence that he or she is, the other sex 2. In boys, preference for cross-dressing or simulating female attire; in girls, insistence on wearing only stereotypical masculine clothing 3. Strong and persistent preferences for cross-sex roles in make-believe play or persistent fantasies of being the other sex 4. Intense desire to participate in the stereotypical games and pastimes of the other sex 5. Strong preference for playmates of the other sex | A. A marked incongruence between one’s experienced/expressed gender and natal gender of at least 6 months in duration, as manifested by at least two of the following:   1. A marked incongruence between one’s experienced/expressed gender and primary and/or secondary sex characteristics (or in young adolescents, the anticipated secondary sex characteristics) 2. A strong desire to be rid of one’s primary and/or secondary sex characteristics because of a marked incongruence with one’s experienced/expressed gender (or in young adolescents, a desire to prevent the development of the anticipated secondary sex characteristics) 3. A strong desire for the primary and/or secondary sex characteristics of the other gender 4. A strong desire to be of the other gender (or some alternative gender different from one’s designated gender) 5. A strong desire to be treated as the other gender (or some alternative gender different from one’s designated gender) 6. A strong conviction that one has the typical feelings and reactions of the other gender (or some alternative gender different from one’s designated gender) |
| B. Persistent discomfort with his or her sex or sense of inappropriateness in the gender role of that sex.  C. The disturbance is not concurrent with a physical intersex condition.  D. The disturbance causes clinically significant distress or impairment in social, occupational, or other important areas of functioning. | B. The condition is associated with clinically significant distress or impairment in social, occupational, or other important areas of functioning. Specify if:   1. The condition exists with a disorder of sex development.   C. The condition is post-transitional, in that the individual has transitioned to full-time living in the desired gender (with or without legalization of gender change) and has undergone (or is preparing to have) at least one sex-related medical procedure or treatment regimen—namely, regular sex hormone treatment or gender reassignment surgery confirming the desired gender (e.g., penectomy, vaginoplasty in natal males; mastectomy or phalloplasty in natal female |

*Table 2. Diagnostic criteria between DSM-IV and DSM-V (13)*

|  | **Key Points** | **Challenges** |
| --- | --- | --- |
| **PMD and PMDD** | PMD and PMDD are linked to an increased risk for suicide.  SSRIs remain first-line treatment, either with continuous dosing or solely in the luteal phase of the menstrual cycle. | There is limited data on TGD-AFAB individuals. |
| **Depression and Anxiety** | Studies suggest a lower rate of depressive symptoms in TGD-AFAB individuals receiving GAHT.  Use of GAHT in TGD individuals over the age of 50 has been associated with reduced anxiety symptoms and lower levels of perceived stress. | Though rates of depression differ by sex amongst cisgender individuals, studies looking at the impact of GAHT on TGD individuals often combine TGD identities without specifying AFAB and AMAB. |
| **PTSD** | TGD individuals report higher rates of exposure to traumatic events. | The high rates of PTSD in the TGD community suggests that there may be varying detection strategies for PTSD or over-pathologizing of stress and discrimination. |
| **Bipolar Disorder** | There is a paucity of data linking GAHT with induction of mania. | Case reports linking exogenous testosterone with the induction of mania primarily involve cisgender men and supratherapeutic doses of testosterone. |
| **Psychosis** | TGD individuals have an increased probability of being diagnosed with a primary psychotic disorder. | Studies indicate potential diagnostic biases in psychotic illnesses amongst TGD individuals. |
| **Gender Dysphoria** | GAHT has not been associated with worsening gender dysphoria. | Studies that demonstrate a negative relationship between GAHT and gender dysphoria suggest that any concurrent increase in dysphoria is related to sociolegal and social factors, rather than the use of GAHT itself. |

*Table 3. Psychiatric and Psychosocial Considerations: Key Points and Challenges*

| **Topic** | **Significance** | **Sample Questions** | **Recommendations** | **Comments** |
| --- | --- | --- | --- | --- |
| **Name** | Utilizing a patient’s chosen name is part of creating a gender-affirming care environment. | *“How would you like me to address you today?”*  *“What is your preferred name?”* | Policies that facilitate a more seamless social transition (i.e.: utilization of a chosen name rather than a legal name) can improve the safety and emotional well-being of TGD patients. | A “deadname” is the name that a TGD or gender-non-binary individual was given at birth but no longer uses. |
| **Pronouns** | Ensuring the use of preferred pronouns is part of creating a gender-affirming care environment. | *“To promote a more inclusive environment for our patients, we like to ask everyone their pronouns. What pronouns do you use?”* | If pronouns are not known or not specifically indicated, consider utilizing gender-neutral pronouns like they/them. | Like deadnaming, utilization of assumed pronouns may be interpreted as an effort to deny or invalidate an individual’s gender identity. |
| **Gender Identity** | Gender identity should not be assumed. | *“How would you describe your gender identity?”*  *“What is your gender identity?”* | Utilize open-ended questions when asking about gender identity.  Do not assume a binary gender identity (man/woman). | Because gender identity refers to ones subjective and internal sense of self, it may not be outwardly visible to others. |
| **Discrimination** | Though discrimination may be experienced as traumatic, it is important to distinguish *trauma* from *discrimination.* | *“Many transgender patients report a history of trauma. Have you ever experienced or witnessed a traumatic event?*  *“Have you ever experienced bias or discrimination based on your gender identity?”* | For patients who report a diagnosis of PTSD, consider utilizing objective measures (e.g., PCL5) or a structured clinical interview to further assess.  Similarly, an objective tool (i.e.: The Trans Discrimination Scale or The Experience of Transgender Discrimination Scale), may be helpful in screening for experiences with anti-TGD bias and discrimination. | Conflation of trauma and discrimination can lead to over-pathologizing of responses to stress. |
| **Menstruation** | While experiences and feelings about menstruation amongst TGD-AFAB individuals are diverse, some may experience an exacerbation of gender dysphoria, or an incongruence between gender identity and perception of their bodies during menses. | *Are you still menstruating?*  *How do you feel when this happens?*  *How has testosterone impacted your menstrual cycle?* | Options for menstrual suppression should be individualized, developmentally appropriate, and consistent with available treatment guidelines. | Engaging TGD-AFB individuals in conversations about menstrual suppression can also provide a forum to explore readiness for gender-affirming treatment and any associated mental health needs. |
| **Pelvic Pain** | Higher rates of pelvic pain have been reported in adult trans cohorts. | *Do you experience any pelvic pain?*  *Is there a correlation between your pelvic pain and your menstrual cycle? What about with orgasm?* | Providers should inquire about pelvic pain, especially in those patients on GAHT, with a personal history of PTSD, experiences of pain with orgasm, and/or continued menstruation.  Pelvic pain may be more common in patients with a history of PTSD. | TGD-AFAB individuals may seek a hysterectomy in response to pelvic pain. |
| **Fertility preservation** | Given the majority of TGD individuals express parental desire, it is vital discuss family planning before initiating testosterone, a known teratogen. | *Have you considered having biological children in the future?* | Review various fertility preservation options, as well as insurance coverage and cost. | Ensuring patients are offered fertility preservation options creates an inclusive space for TGD people to consider family planning. |
| **Contraception** | The irregular nature of ovulation while on GAHT makes pregnancy harder to anticipate, increasing the risk for unplanned pregnancies amongst TGD-AFAB individuals. | *“What methods are you currently using for contraception or pregnancy prevention?”* | Do not assume a patients sexual identity or sexual practices.  Providers should review various contraception options that are available to TGD-AFAB individuals. | A common misconception amongst TGD-AFAB patients and providers is that testosterone provides reliable contraception. |

*Table 4. The Clinical Evaluation of Transgender and Gender Diverse Menstruators: Relevant Considerations & Recommendations for Patient Assessment*
